# High TCR Degeneracy Enhances Antiviral Efficacy of HTLV-1-Specific CTLs by Targeting Variant Viruses in HAM Patients

**DOI:** 10.3390/ijms26146602

**Published:** 2025-07-10

**Authors:** Ryuji Kubota, Kousuke Hanada, Mineki Saito, Mika Dozono, Satoshi Nozuma, Hiroshi Takashima

**Affiliations:** 1 Division of Neuroimmunology, Joint Research Center for Human Retrovirus Infection, Kagoshima University, 8-35-1 Sakuragaoka, Kagoshima 890-8544, Japan; 2 Department of Bioscience and Bioinformatics, Kyushu Institute of Technology, 680-4 Kawazu, Iizuka-shi 820-8502, Japan; kohanada@bio.kyutech.ac.jp; 3 Department of Microbiology, Kawasaki Medical School, 577 Matsushima, Kurashiki 701-0192, Japan; mineki@med.kawasaki-m.ac.jp; 4 Department of Neurology and Geriatrics, Kagoshima University, 8-35-1 Sakuragaoka, Kagoshima 890-8520, Japan; k3783253@kadai.jp (M.D.); snozuma@m2.kufm.kagoshima-u.ac.jp (S.N.); thiroshi@m3.kufm.kagoshima-u.ac.jp (H.T.)

**Keywords:** HTLV-1, HTLV-1-associated myelopathy, T-cell receptor degeneracy, cytotoxic T lymphocytes, viral load, epitope variants

## Abstract

T-cell receptors (TCRs) exhibit degeneracy, enabling individual TCRs to recognize multiple altered peptide ligands (APLs) derived from a single cognate antigen. This characteristic has been involved in the pathogenesis of autoimmune diseases through cross-reactivity between microbial and self-antigens. Cytotoxic T lymphocytes (CTLs), which recognize peptide–MHC class I complexes via TCRs, play a critical role in the immune response against viral infections. However, the extent to which TCR degeneracy within a population of virus-specific CTLs contributes to effective viral control remains poorly understood. In this study, we investigated the magnitude and functional relevance of TCR degeneracy in CTLs targeting an immunodominant epitope of human T-cell leukemia virus type 1 (HTLV-1) in patients with HTLV-1-associated myelopathy (HAM). Using peripheral blood mononuclear cells (PBMCs) from these patients, we quantified TCR degeneracy at the population level by comparing CTL responses to a panel of APLs with responses to the cognate epitope. Our findings demonstrated that increased TCR degeneracy, particularly at the primary TCR contact residue at position 5 of the antigen, was inversely correlated with HTLV-1 proviral load (*p* = 0.038, R = −0.40), despite similar functional avidity across patient-derived CTLs. Viral sequencing further revealed that CTLs with high TCR degeneracy exerted stronger selective pressure on the virus, as indicated by a higher frequency of nonsynonymous substitutions within the epitope-encoding region in patients with highly degenerate TCR repertoires. Moreover, TCR degeneracy was positively correlated with the recognition rate of epitope variants (*p* = 0.018, R = 0.76), suggesting that CTLs with high TCR degeneracy exhibited enhanced recognition of naturally occurring epitope variants compared to those with low TCR degeneracy. Taken together, these results suggest that virus-specific CTLs with high TCR degeneracy possess superior antiviral capacity, characterized by broadened epitope recognition and more effective suppression of HTLV-1 infection. To our knowledge, this is the first study to systematically quantify TCR degeneracy in HTLV-1-specific CTLs and evaluate its contribution to viral control in HAM patients. These findings establish TCR degeneracy as a critical determinant of antiviral efficacy and provide a novel immunological insight into the mechanisms of viral suppression in chronic HTLV-1 infection.

## 1. Introduction

Antigenic stimulation induces the expansion of diverse T-cell clones with high specificity for their cognate antigens, including viral antigens such as those derived from human immunodeficiency virus (HIV), cytomegalovirus (CMV), and influenza virus, as well as autoantigens such as myelin basic protein [1,2,3,4]. While T-cell receptors (TCRs) were once thought to recognize a single antigen with high specificity, evidence shows that a single TCR can recognize both its cognate peptide and various altered peptide ligands (APLs) [5,6]. This phenomenon, termed TCR degeneracy [7], has been extensively studied using antigen-specific T-cell clones or T-cell lines [8,9], particularly in the context of autoimmunity, where cross-recognition of microbial and self-antigens may contribute to pathogenesis [2]. TCR degeneracy is also observed in viral infections such as HIV and CMV. For example, a public HIV-specific TCR has been shown to cross-recognize multiple epitope variants using yeast display techniques [10]. In other studies, HIV-specific CD8+ T cells have been found to cross-react with diverse HIV-1 epitopes, and microbial peptides that cross-react with HIV epitopes have been shown to modulate CTL antiviral activity [11,12]. These findings highlight the broader relevance of TCR degeneracy in antiviral immunity. However, the physiological significance of TCR degeneracy at the population level of antigen-specific T cells remains largely unexplored. In particular, it remains unclear whether this degeneracy enhances the capacity of virus-specific cytotoxic T lymphocytes (CTLs) to control viral infection in vivo.

CD8+ virus-specific CTLs are critical effectors in antiviral immunity, and several of their characteristics—such as frequency, antigenic breadth, and functional avidity—have been associated with viral control. For example, in HIV-1 infection, the frequency of virus-specific CTLs negatively correlates with plasma viral load [13]. Furthermore, the number of recognized CTL epitopes and the avidity of CTL responses have been linked to the efficiency of target cell lysis and overall antiviral efficacy [14,15]. Nonetheless, the impact of TCR degeneracy on viral control remains poorly understood. In a previous study, we observed that the frequency and degeneracy of virus-specific CTLs increased following viral replication and were associated with a subsequent decline in viral load [16], suggesting a possible role for TCR degeneracy in antiviral defense.

Human T-cell leukemia virus type 1 (HTLV-1) is a human retrovirus that preferentially infects CD4+ T lymphocytes in vivo [17]. Although the majority of infected individuals remain asymptomatic, approximately 1% develop HTLV-1-associated diseases, including the neuroinflammatory disorder HTLV-1-associated myelopathy (HAM) and adult T-cell leukemia/lymphoma [18,19,20]. HAM is characterized by chronic inflammation of the spinal cord and prominent perivascular infiltration of mononuclear cells [21]. Clinically, patients present with spastic paraparesis, sphincter dysfunction, and sensory disturbances in the lower extremities [22]. In these patients, HTLV-1-specific CD8+ CTLs are elevated in both peripheral blood mononuclear cells (PBMCs) [23,24,25] and cerebrospinal fluid (CSF), with their frequency being even higher in the CSF than in peripheral blood [26]. Moreover, an extremely high frequency of HTLV-1—specific CTLs has been reported in the spinal cords of HAM patients, indicating strong local immune responses within the central nervous system [27]. CTLs primarily target the HTLV-1 Tax protein, with the Tax 11–19 epitope recognized as an immunodominant peptide in patients expressing HLA-A*02 [28]. This epitope has been extensively studied and is known to exhibit strong binding affinity to the corresponding TCR [29], eliciting robust CTL responses. In PBMCs from HAM patients, the frequency of Tax 11–19–specific CTLs is remarkably high, accounting for an average of 1.9% of CD8+ T cells [16]. Despite these robust CTL responses, HAM patients typically exhibit higher HTLV-1 proviral loads (PVLs) than asymptomatic HTLV-1 carriers, and PVL is a known risk factor for disease development and progression [23]. Thus, reduction of PVL is a critical therapeutic objective, yet no effective treatment to achieve this has been established. Given the central role of CTLs in viral control, detailed analyses of CTL properties—including TCR degeneracy—that may influence PVL are essential for the development of effective therapeutic strategies.

In the present study, we investigated HTLV-1 Tax 11–19-specific CTLs in HAM patients to evaluate their frequency, TCR degeneracy, and TCR structural diversity. We also measured HTLV-1 PVL and analyzed naturally occurring viral variants in PBMCs. These comprehensive analyses allowed us to examine the relationship between CTL properties and viral load, thereby providing insights into the role of TCR degeneracy in antiviral immunity.

## 2. Results

### 2.1. Recognition of Altered Peptide Ligands (APLs) by HTLV-1 Tax 11–19–Specific CD8+ CTLs

**Figure 1:**
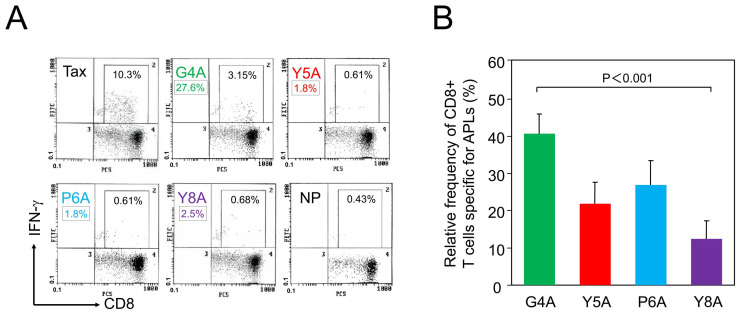
Recognition of altered peptide ligands (APLs) by HTLV-1 Tax 11–19-specific CD8+ cytotoxic T lymphocytes (CTLs). HTLV-1 Tax 11–19 and APL-specific CD8+ CTLs were detected in PBMCs from 24 patients with HAM. (**A**) Representative flow cytometry data from patient H1. PBMCs were co-cultured for 6 h with HLA-A*02 + antigen-presenting cells pulsed with either the cognate peptide (Tax 11–19), an APL, or no-peptide (NP), in the presence of brefeldin A. Cells were stained with anti-CD8 and anti–interferon (IFN)-γ antibodies. CD8+ cells were gated, and the percentages indicate the frequency of IFN-γ + cells within the CD8+ cell population. The mean background frequency under the no-peptide (NP) condition was 0.37 ± 0.21% (mean ± SD) across all 24 patients. Relative recognition of each APL to the HTLV-1 Tax 11–19 epitope was calculated as described in Materials and Methods. For example, in patient H1, the relative frequency of G4A-specific CTLs was calculated as follows: (3.15 − 0.43)/(10.3 − 0.43) × 100 = 27.6%. Using the same formula, the relative frequencies for Y5A, P6A, and Y8A were 1.8%, 1.8%, and 2.5%, respectively. These values are indicated as boxed numbers beneath each corresponding APL name. (**B**) Summary of relative frequencies of APL-specific CTLs, expressed as a percentage of the response to the cognate peptide, in all 24 patients. Columns represent mean values, and error bars indicate the standard error of the mean. Y5A and Y8A, corresponding to primary and secondary TCR contact residues, respectively, were recognized less frequently than G4A and P6A.

To assess TCR degeneracy, we employed APLs generated by alanine substitution at specific TCR contact sites within the cognate HTLV-1 Tax 11–19 epitope. Alanine substitution is widely used because it minimally affects peptide conformation and MHC binding, thereby allowing precise evaluation of individual residues in TCR recognition. This approach is a standard method for analyzing TCR degeneracy [30]. We measured the frequency of CD8+ T cells specific for the cognate peptide and its alanine-substituted variants to evaluate TCR recognition breadth. Four APLs—G4A, Y5A, P6A, and Y8A—were designed by substituting alanine at positions 4, 5, 6, and 8, respectively, which correspond to primary (positions 4–6) or secondary (position 8) TCR contact residues [29]. Representative flow cytometric analysis from patient H1 demonstrated distinct populations of CD8+ cells reactive to each APL (Figure 1A). A summary of relative frequencies of APL-specific CTLs, expressed as percentages relative to the response to the cognate peptide, is shown for all 24 HAM patients in Figure 1B. Notably, the relative frequencies of CTLs specific for the Y5A and Y8A variants were markedly lower than those for G4A and P6A.

### 2.2. TCR Degeneracy Correlates Negatively with HTLV-1 PVL

**Figure 2:**
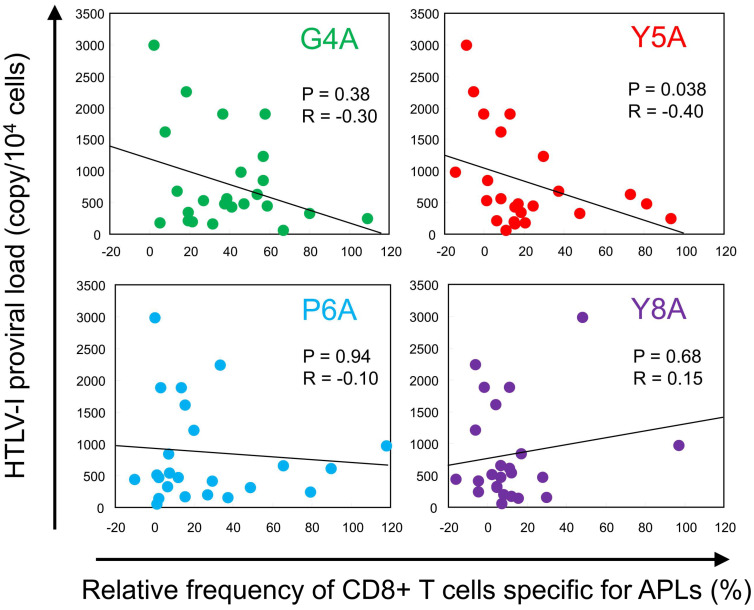
Inverse correlation between TCR degeneracy and HTLV-1 proviral load (PVL). HTLV-1 PVL was plotted against the relative frequency of CTL responses to each APL. Each dot represents an individual patient. A negative correlation was observed for all APLs except Y8A. Notably, a statistically significant inverse correlation was identified between Y5A recognition and PVL (*p* = 0.038, R = −0.40; Spearman’s rank correlation test).

To explore the relationship between TCR degeneracy and viral burden, we plotted HTLV-1 PVL against the relative frequency of CD8+ T-cell responses to each APL (Figure 2). For all APLs except Y8A, we observed negative correlations between PVL and the magnitude of CTL responses to the APLs. The inverse correlation was statistically significant for the Y5A-specific response (*p* = 0.038, R = −0.40). To evaluate cumulative responsiveness, we calculated the mean relative recognition across all APLs and assessed its association with PVL. However, no significant correlation was detected (*p* = 0.27). Additionally, the overall frequency of Tax 11–19-specific CTLs did not correlate with PVL (*p* = 0.38).

### 2.3. Functional Avidity of CTLs Is Comparable Among Patients

**Figure 3:**
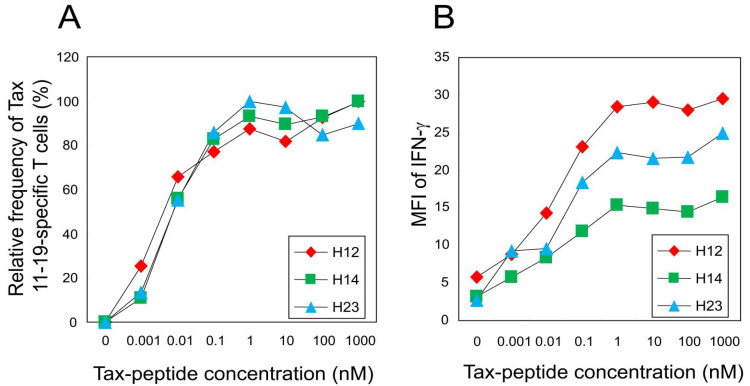
Functional avidity of HTLV-1 Tax 11–19-specific CTLs is comparable among patients with differing TCR degeneracy. Three HAM patients (H12, H14, and H23) with distinct levels of Y5A recognition (38.0%, 8.9%, and 73.7%, respectively) were analyzed for CTL functional avidity. (**A**) Peptide titration curves of IFN-γ + CD8+ T cells are normalized to the maximal response observed in each patient. The peptide concentrations required to elicit 50% of the maximal IFN-γ + cell frequency are 6.5 pM (H12), 8.8 pM (H14), and 8.9 pM (H23). (**B**) Mean fluorescence intensity (MFI) of IFN-γ production is plotted against peptide concentration. The concentrations required to achieve 50% of the maximal MFI are 44.3 pM, 48.2 pM, and 52.9 pM for H12, H14, and H23, respectively.

To evaluate whether differences in TCR degeneracy could be attributed to variations in functional T-cell avidity, we selected three representative patients (H12, H14, and H23) who exhibited substantial inter-individual variation in both the frequency of Tax 11–19–specific CTLs (19.5%, 3.3%, and 5.5%, respectively) and relative Y5A recognition (38.0%, 8.9%, and 73.7%, respectively; Table 1). Despite these differences, CTLs from all three patients displayed comparable dose—response characteristics when stimulated with titrated concentrations of the cognate peptide (Figure 3A). The peptide concentrations required to elicit 50% of maximal interferon (IFN)-γ + cell frequency were 6.5 pM (H12), 8.8 pM (H14), and 8.9 pM (H23). Similarly, the concentrations required for 50% maximal IFN-γ mean fluorescence intensity (MFI) were 44.3 pM (H12), 48.2 pM (H14), and 52.9 pM (H23) (Figure 3B). These findings indicate that CTLs across patients possess similar functional avidity, suggesting that differences in TCR degeneracy are not attributable to differences in antigen sensitivity.

### 2.4. Correlation Between TCR Structural Diversity and PVL

**Figure 4:**
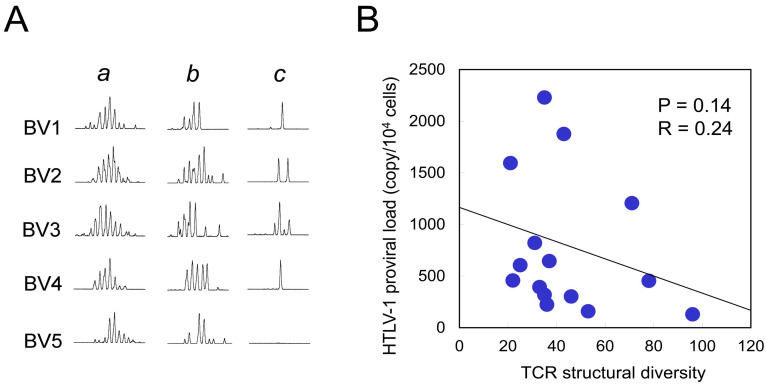
Correlation between TCR structural diversity and PVL. (**A**) Representative TCR BV CDR3 spectratyping results are shown for five TCR BV families (BV1–BV5). Lane a: 1 × 10^5^ PBMCs from a healthy control, displaying a Gaussian distribution indicative of polyclonal TCR repertoires. Lane b: 1 × 10^4^ PBMCs from the same healthy control, showing partial loss of peak distribution due to reduced input. Lane c: 1 × 10^4^ purified Tax 11–19-specific CTLs from a HAM patient, exhibiting an oligoclonal pattern with reduced peak diversity. Due to the limited availability of HTLV-1 Tax 11–19-specific CTLs, a cell input of 1 × 10^4^ was used for subsequent analyses. TCR structural diversity was quantified by summing the number of distinct CDR3 length peaks across all 26 TCR BV families. The mean total peak count in Tax 11–19-specific CTLs from 15 HAM patients was 44.1 ± 21.7 (mean ± SD), compared to 106 peaks in control PBMCs (lane b), indicating reduced TCR diversity in the patient group. (**B**) TCR structural diversity, measured by the total number of CDR3 length peaks, is plotted against HTLV-1 PVL, revealing a negative correlation that did not reach statistical significance (*p* = 0.14).

To assess whether TCR structural diversity is associated with HTLV-1 PVL, we performed TCR BV complementarity-determining region 3 (CDR3) spectratyping. Representative profiles from TCR BV1 to BV5 among 26 analyzed TCR BV families are shown in Figure 4A. In a healthy control, the analysis of 1 × 10^5^ PBMCs yielded a symmetrical Gaussian distribution (Figure 4A, lane a), consistent with a polyclonal T-cell repertoire. In contrast, using a reduced input of 1 × 10^4^ PBMCs led to partial loss of symmetry (lane b), which served as a control for low cell number. Given the scarcity of HTLV-1-specific CTLs, we employed this input level for subsequent analyses. We examined HTLV-1 Tax 11–19-specific CTLs isolated from 15 HLA-A*02-positive HAM patients. In contrast to healthy controls (lane b), these patients exhibited a markedly diminished number of CDR3 peaks (lane c), indicative of oligoclonal or monoclonal expansion. The total number of CDR3 length peaks across all TCR BV families was 106 in the healthy control at the 1 × 10^4^ cell input level, compared to 44.1 ± 21.7 (mean ± SD) in HAM patients (N = 15). This total peak count—used as a proxy for TCR structural diversity—showed a negative correlation with HTLV-1 PVL, although the association did not reach statistical significance (*p* = 0.14, R = 0.24; Figure 4B). In contrast, no correlation was observed between TCR structural diversity and TCR degeneracy, as assessed using Y5A recognition (*p* = 0.45, R = 0.02).

### 2.5. CTLs with High TCR Degeneracy Exert Strong Antiviral Pressure

To evaluate whether TCR degeneracy influences the magnitude of antiviral selective pressure, we analyzed the HTLV-1 gene sequence encoding the immunodominant Tax 11–19 epitope and calculated the difference between nonsynonymous and synonymous substitution rates (Dn-Ds). This approach has previously been used to assess CTL-mediated immune selection [31]. We sequenced the Tax 11–19 region in 18 HLA-A*02-positive HAM patients (Table 1) and stratified them into high and low TCR degeneracy groups based on the mean relative frequency of CTL responses to each APL.

**Figure 5:**
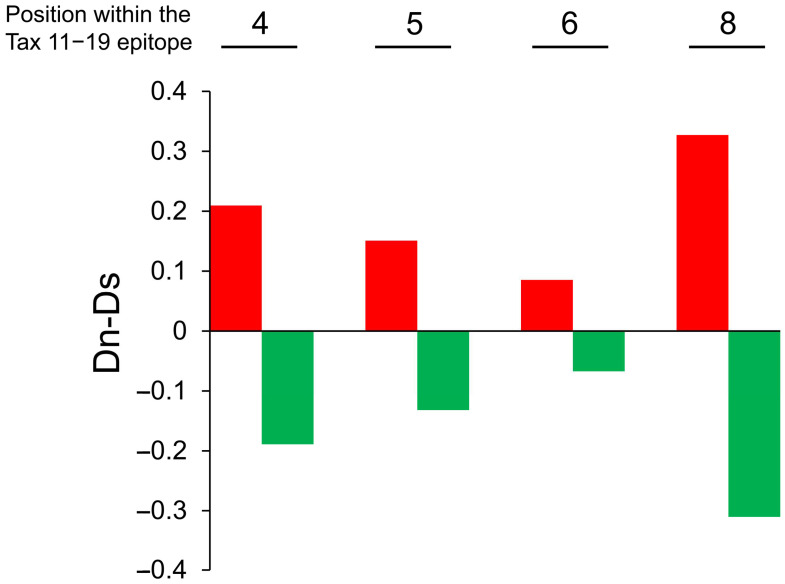
Increased nonsynonymous substitutions in the HTLV-1 Tax 11–19 epitope in patients with high TCR degeneracy. Eighteen HLA-A*02-positive HAM patients were stratified into high and low TCR degeneracy groups based on the mean relative recognition of APLs at each TCR contact residue. Group classifications are as follows: Position 4: high degeneracy (patient H3, H7, H8, H13, H15, H19, H20, H23, H24), and low degeneracy (H2, H6, H9, H10, H11, H12, H14, H16, H22). Position 5: high degeneracy (H7, H8, H9, H11, H12, H16, H20, H23, H24), and low degeneracy (H2, H3, H6, H10, H13, H14, H15, H19, H22). Position 6: high degeneracy (H6, H7, H12, H15, H16, H19, H20, H23, H24), and low degeneracy (H2, H3, H8, H9, H10, H11, H13, H14, H22). Position 8: high degeneracy (H2, H3, H8, H9, H10, H16, H19, H22, H23), and low degeneracy (H6, H7, H11, H12, H13, H14, H15, H20, H24). Sequence analysis of the Tax 11–19 region was performed to calculate Dn-Ds values at amino acid positions 4, 5, 6, and 8. In the high-degeneracy group (red bars), Dn-Ds values are consistently positive, indicating stronger CTL-mediated selective pressure. In contrast, the low-degeneracy group (green bars) exhibits consistently negative values, suggesting weaker immune pressure.

Dn-Ds values at amino acid positions 4, 5, 6, and 8 within the HTLV-1 Tax 11–19 epitope are shown for each group in Figure 5, with red and green bars representing the high- and low-degeneracy groups, respectively. Dn-Ds values were consistently greater than zero across all examined residues in the high-degeneracy group, whereas values in the low-degeneracy group were consistently below zero, indicating stronger CTL-mediated selective pressure in the former.

Additionally, the total number of nonsynonymous (Nn) and synonymous (Ns) substitutions differed significantly between the two groups, with a higher frequency of substitutions observed in the high-degeneracy group (*p* = 0.018; Table 2). These findings support the conclusion that CTLs with high TCR degeneracy exert greater antiviral pressure on HTLV-1.

Eighteen patients with HAM were stratified into two groups based on the mean value of TCR degeneracy, as determined by the relative recognition of APLs. The total number of nonsynonymous (Nn) and synonymous (Ns) substitutions within the HTLV-1 Tax gene region encoding the Tax 11–19 epitope is quantified for each group. The high TCR degeneracy group exhibits a significantly greater number of nonsynonymous substitutions compared to the low-degeneracy group (*p* = 0.018, two-tailed chi-square test), suggesting that individuals with high TCR degeneracy exert stronger CTL-mediated immune pressure on the virus.

### 2.6. TCR Degeneracy Enhances Recognition of Naturally Occurring Variant Epitopes

**Figure 6:**
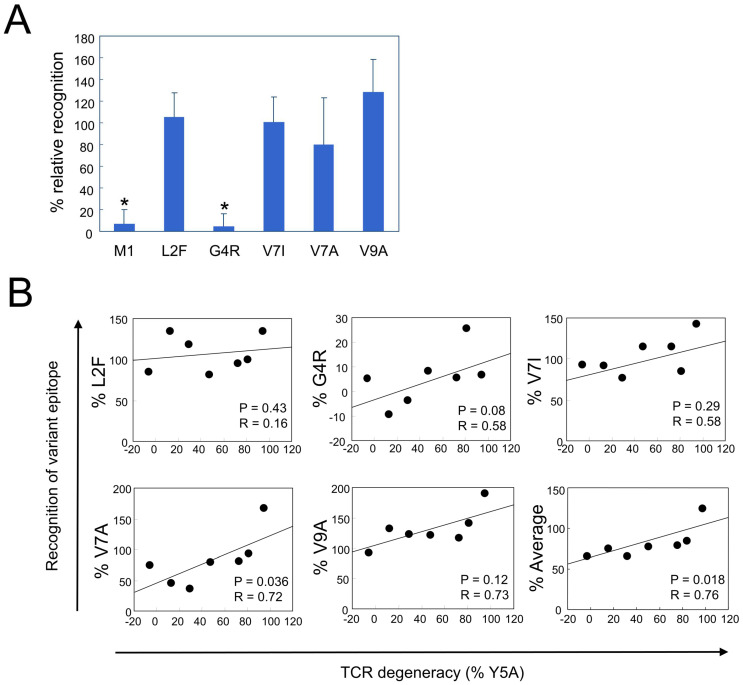
Positive correlation between TCR degeneracy and recognition of naturally occurring HTLV-1 epitope variants. Five naturally occurring variants of the HTLV-1 Tax 11–19 epitope—L2F, G4R, V7I, V7A, and V9A—were identified (Table 1), and CTL responses to each variant were assessed. (**A**) Relative recognition of each variant is expressed as a percentage of the response to the cognate Tax 11–19 peptide. Error bars represent the SD. Asterisks (*) indicate statistically significant differences (*p* < 0.001) compared with the recognition levels of L2F, V7I, V7A, and V9A. (**B**) Relative recognition of individual variants, expressed as a percentage of the response to the cognate HTLV-1 Tax 11–19 peptide, is plotted against TCR degeneracy, defined by the CTL response to the Y5A APL. A significant positive correlation is observed for the V7A variant (*p* = 0.036, R = 0.72). The bottom-right panel summarizes the average recognition across all five variants, which also correlated significantly with TCR degeneracy (*p* = 0.018, R = 0.76).

We next investigated whether TCR degeneracy facilitates the recognition of naturally occurring epitope variants. Sequence analysis of the HTLV-1 Tax gene in PBMCs from 18 HLA-A*02-positive HAM patients identified amino acid substitution within the HTLV-1 Tax 11–19 epitope in 9 patients. In total, 17 unique substitutions were found in a total of 818 sequences, corresponding to a variant frequency of 2.08%. Among the identified variants—L2F, G4R, V7I, V7A, and V9A—the most frequent was G4R, accounting for 12 of the 17 variant sequences (70.6%, Table 1). Patient-specific variant frequencies ranged from 0% to 10.4%, with a mean of 2.1%. To evaluate CTL recognition of these naturally occurring variants, we synthesized variant peptides and conducted functional assays using PBMCs from 7 of 9 patients for whom sufficient cell material was available. The L2F, V7I, and V7A variants elicited CTL responses comparable to those of the cognate peptide. In contrast, the G4R variant was minimally recognized, whereas V9A elicited a stronger response than the cognate epitope (Figure 6A). We then examined whether TCR degeneracy influences the recognition of these variants. Given that position 5 is a primary TCR contact site and that Y5A recognition was inversely correlated with PVL, Y5A responsiveness was used as a surrogate marker of TCR degeneracy. Among the five tested variants, recognition levels correlated positively with TCR degeneracy, with a statistically significant association observed for the V7A variant (*p* = 0.036, R = 0.72; Figure 6B). Furthermore, the average relative recognition across all variants also correlated significantly with TCR degeneracy (*p* = 0.018, R = 0.76). These findings suggest that CTLs with high TCR degeneracy are better equipped to recognize and respond to naturally emerging epitope variants, thereby contributing to more effective immune surveillance and control of HTLV-1 infection.

## 3. Discussion

In this study, we analyzed the frequency of HTLV-1 Tax 11–19-specific CD8+ CTLs, their TCR degeneracy and structural diversity, HTLV-1 PVL, and viral epitope variants in PBMCs from patients with HAM. We then examined how these CTL characteristics relate to proviral burden. Among the parameters evaluated, only TCR degeneracy, measured as the relative recognition of the Y5A APL, was inversely correlated with PVL. In contrast, CTL frequency, IFN-γ production (quantified by mean fluorescence intensity), and TCR structural diversity showed no significant association with viral load. These findings suggest that population-level TCR degeneracy plays a key role in CTL-mediated viral control in vivo. In addition to its inverse correlation with PVL, high TCR degeneracy was associated with a higher frequency of nonsynonymous (Dn) relative to synonymous (Ds) substitutions within the viral epitope, as well as elevated Sn values (the number of synonymous sites) across the viral genome. These signatures of immune-driven selection suggest that CTLs with high TCR degeneracy exert greater antiviral pressure on HTLV-1 compared to those with limited degeneracy. Accordingly, patients with high TCR degeneracy may be more effective at controlling HTLV-1 replication in vivo. In Epstein-Barr virus infection, cross-reactive memory CD8+ T cells have demonstrated broad peptide reactivity—even across distinct HLA alleles—highlighting degenerate peptide recognition as a mechanism for sustained antiviral control [32]. Similarly, a fine specificity analysis of an HLA-A*02-restricted HIV Gag epitope revealed that although most of the 171 tested variants could bind MHC, one-third were recognized by T cells, a result attributed to structural constraints shaping TCR cross-reactivity [33]. Notably, T cells that cross-recognized these variants exhibited enhanced control of HIV replication in vivo, underscoring the functional relevance of TCR degeneracy. Our findings are consistent with these studies, further supporting the importance of TCR degeneracy in antiviral immunity.

Notably, Y5A is a variant of the HTLV-1 Tax 11–19 epitope in which tyrosine at position 5, the primary TCR contact residue, is replaced by alanine. This substitution disrupts TCR recognition without affecting the peptide’s binding affinity for HLA-A*02 molecules [29,34]. Tyrosine, with its bulky aromatic side chain, can form hydrogen bonds that are critical for stabilizing the interface between the TCR and the peptide–MHC (pMHC) complex. In contrast, alanine, with only a small nonpolar methyl group, lacks these interactive capacities. Therefore, substituting Y5 with alanine likely disrupts essential molecular contacts required for effective TCR recognition. This interpretation is supported by structural studies of TCR-pMHC complexes. The crystal structure of a human TCR bound to an HLA-A*02:01 molecule presenting HTLV-1 Tax 11–19 peptide revealed that the central residues—particularly position 5 (P5)—are often deeply embedded within a pocket formed by the hypervariable CDR3 loops of the TCR, especially CDR3β [29]. In this configuration, the side chain of the P5 residue directly contacts the base of the CDR3β loop, functioning as a structural “hot spot” essential for productive TCR engagement. Residues at positions 4 to 6 of the Tax 11–19 epitope collectively form the primary TCR contact interface. In our cohort, CTL recognition of the Y5A variant was consistently lower than that of other APLs with substitutions at key contact residues (e.g., G4A and P6A), emphasizing the functional importance of position 5 (Figure 1B). Furthermore, the degree of Y5A recognition by T cells was significantly negatively correlated with the PVL (Figure 2). These observations support the utility of APLs with substitutions at key TCR contact residues—particularly at position 5—as sensitive tools for evaluating TCR degeneracy.

To elucidate the mechanism by which TCR degeneracy enhances antiviral efficacy, we investigated whether it is associated with CTL functional avidity. Our data demonstrated comparable levels of functional avidity across three patients, regardless of their degree of TCR degeneracy (Figure 3). These findings suggest that variation in TCR degeneracy may not influence CTL avidity in our HTLV-1 cohort; however, they do not imply that TCR degeneracy is inherently superior to functional avidity in controlling viral infection [35]. Rather, they indicate that TCR degeneracy serves as an independent determinant of CTL effectiveness [36]. This interpretation is conceptually supported by the framework, in which extensive TCR cross-reactivity is viewed as a fundamental immunological strategy to recognize a vast array of potential peptide variants using a limited number of TCR clonotypes [37]. Based on this concept, we hypothesized that highly degenerate TCRs would be better suited to recognize epitope variants. Indeed, CTLs from patients with high TCR degeneracy exhibited significantly enhanced recognition of naturally occurring variants of the HTLV-1 Tax 11–19 epitope. This broadened recognition likely constrains the expansion of viral escape mutants, thereby contributing to the observed reduction in PVL. Taken together, these results suggest that when CTLs possess comparable levels of avidity, it is their ability to accommodate epitope variation—through TCR degeneracy—that enables more effective antiviral surveillance and containment of viral escape. This functional breadth appears to be a key factor in antiviral efficacy in the context of ongoing viral evolution.

HTLV-1 establishes lifelong infection and can lead to chronic inflammation in various organs, including the spinal cord, skeletal muscles, eyes, lungs, and joints [38,39,40]. While viral protein expression is typically low in circulating PBMCs, it becomes detectable upon short-term ex vivo culture [41]. HAM patients exhibit elevated titers of anti-HTLV-1 IgM antibodies and a higher frequency of Tax-specific CTLs compared with asymptomatic HTLV-1 carriers [24,42], suggesting ongoing antigenic stimulation and persistent immune activation. The presence of multiple epitope variants and the increased frequency of nonsynonymous substitutions in the high TCR degeneracy group further support the notion of active viral replication in these individuals. These findings align with our previous report showing that TCR degeneracy in HTLV-1-specific CTLs increases during periods of heightened viral replication [16]. Thus, the expansion of CTLs with high TCR degeneracy may reflect both sustained antigen exposure and adaptive immune responses to ongoing viral evolution.

Viral load is governed by both viral and host factors. On the viral side, replication kinetics, epitope variability, and the level of antigen expression in infected cells are critical determinants. Host-related factors include CTL frequency, effector function (e.g., cytotoxicity and cytokine production), epitope breadth, and TCR avidity [13,15]. Our findings add a new dimension to this framework by identifying TCR degeneracy in virus-specific CTLs as a key factor in the control of HTLV-1. By enabling recognition of both canonical and variant epitopes, CTLs with high TCR degeneracy are likely to suppress viral replication more effectively than clonotypes with narrow specificity. These results have important therapeutic implications, suggesting that the adoptive transfer of CTLs with broad TCR recognition capabilities may enhance viral suppression and reduce HTLV-1 PVL more efficiently than strategies based solely on high-avidity or high-frequency CTLs.

In conclusion, we systematically quantified TCR degeneracy in HTLV-1-specific CTLs and demonstrated its significant association with the control of viral replication in HAM patients. Our findings reveal that TCR degeneracy enables broader recognition of epitope variants, serving as an independent determinant of antiviral efficacy alongside functional avidity. These insights highlight TCR degeneracy as a novel immunological correlate of viral control and suggest its potential as a therapeutic target for enhancing immune responses against HTLV-1.

## 4. Materials and Methods

### 4.1. Ethics Statement

This study was conducted in accordance with the principles outlined in the Declaration of Helsinki and was approved by the Institutional Ethics Committee of Kagoshima University. Written informed consent was obtained from all participants for the collection and analysis of peripheral blood samples.

### 4.2. Study Subjects

A total of 24 patients diagnosed with HAM, according to the World Health Organization (WHO) diagnostic criteria and confirmed to be positive for the HLA-A*02 allele, were enrolled in this study. PBMCs were isolated from whole blood samples, and HLA-A*02 typing was performed as previously described [43]. The clinical characteristics of the HAM patients are summarized in Supplementary Table S1, including a mean age of 57.3 ± 11.3 (mean ± SD) years, a PVL of 793.8 ± 772.3 copies/10^4^ PBMCs, and a frequency of HTLV-1 Tax 11–19-specific CD8+ T cells among CD8+ T cells of 3.1 ± 4.2%. The cohort included 17 female and 7 male patients. These 24 patients were analyzed for TCR degeneracy. Due to sample limitations, HTLV-1 gene sequencing was performed in 16 patients, and TCR CDR3 spectratyping was conducted in 15 patients.

### 4.3. Synthetic Peptides

The HTLV-1 Tax 11–19 epitope (LLFGYPVYV), a well-characterized immunodominant peptide presented by HLA-A*02 to HTLV-1-specific CTLs [24], was synthesized along with a panel of altered peptide ligands (APLs) incorporating single L-alanine substitutions at known TCR contact residues. Residues at positions 4, 5, and 6 serve as primary TCR contact sites, with position 5 acting as the major contact point; residue 8 is considered a secondary contact site [29]. The resulting APLs—designated G4A, Y5A, P6A, and Y8A—were synthesized at >90% purity. An HLA-A*02-restricted influenza matrix protein peptide (M1; GILGFVFTL) was used as a specificity control. Additionally, five naturally occurring HTLV-1 Tax 11–19 epitope variants (L2F, G4R, V7I, V7A, and V9A) were synthesized.

### 4.4. Assessment of TCR Degeneracy by CTL Recognition of APLs

TCR degeneracy was evaluated by measuring CTL recognition of each APL relative to the cognate Tax 11–19 peptide, following a modified protocol described previously [16]. Briefly, HLA-A*02:01–transfected Hmy2.C1R cells (Hmy-A2) were pulsed with 1 μM of either Tax 11–19 or an APL for 1 h at 37 °C. Subsequently, 5 × 10^5^ PBMCs were co-cultured with an equal number of peptide-pulsed Hmy-A2 cells for 6 h in the presence of brefeldin A (Sigma-Aldrich, Tokyo, Japan). The typical incubation time for intracellular cytokine detection ranges from 6 to 12 h [44], and IFN-γ expression in HTLV-1-specific CD8+ T cells has been previously detected after 6 h of stimulation [16,45]. Based on these findings, we adopted a 6-hour stimulation period. After stimulation, cells were stained for surface CD8 and intracellular IFN-γ, and analyzed using flow cytometry using an EPICS XL cytometer (Beckman Coulter, Tokyo, Japan). The percentage of IFN-γ + CD8+ cells within the total CD8+ cell population was quantified. CTL frequency was calculated by subtracting the background frequency of IFN-γ + CD8+ cells (no-peptide condition) from the frequency observed in peptide-stimulated conditions. TCR degeneracy was quantified as the relative frequency (%) using the following formula:

(% relative frequency) = (Frequency of IFN-γ + CD8+ cells with APL-background)/(Frequency of IFN-γ + CD8+ cells with Tax 11–19 − background) × 100

Here, background refers to the frequency of IFN-γ + CD8+ T cells in the absence of peptide stimulation, as indicated by the “no-peptide” (NP) condition in Figure 1A.

### 4.5. Functional T-Cell Avidity Assay

To evaluate functional T-cell avidity, we selected three HAM patients (H12, H14, and H23) who exhibited distinct levels of CTL frequency and TCR degeneracy (Table 1) [16]. These patients demonstrated substantial inter-individual variation in both the frequency of Tax 11–19-specific CTLs (19.5%, 3.3%, and 5.5%, respectively) and the TCR degeneracy (38.0%, 8.9%, and 73.7%, respectively). Tax 11–19-specific CTL responses were measured at various peptide concentrations and normalized to the maximal IFN-γ response for each individual. The peptide concentration required to elicit 50% of the maximal IFN-γ response (EC50) was calculated and used as an index of functional avidity.

### 4.6. TCR BV CDR3 Spectratyping of Tax 11–19–Specific CD8+ CTLs

Tax 11–19-specific CD8+ CTLs were isolated from the PBMCs of 15 HAM patients using phycoerythrin-labeled HLA-A*02:01/Tax 11–19 tetramers (MBL, Nagoya, Japan), followed by magnetic separation with anti-phycoerythrin antibody-conjugated microbeads (Miltenyi Biotec, Tokyo, Japan), achieving a purity of >95%. Total RNA was extracted from 1 × 10^4^ purified CTLs and reverse-transcribed into cDNA. TCR BV CDR3 spectratyping was conducted using a panel of 26 TCR BV-specific primers and a 6-carboxyfluorescein-labeled constant region primer, as previously described [46]. PCR products were separated via capillary electrophoresis, and fluorescence signals were analyzed using GeneScan (version 1.0) software. TCR structural diversity was evaluated by counting the number of distinct CDR3 length peaks within each TCR BV family and summing them across all families [47].

### 4.7. Quantification of HTLV-1 PVL

Genomic DNA was extracted from the PBMCs of all 24 patients. HTLV-1 PVL was quantified using real-time PCR, following a previously established protocol [23].

### 4.8. Sequence Analysis of the HTLV-1 Tax Gene

The nucleotide sequence encoding the HTLV-1 Tax 11–19 epitope was analyzed in 18 patients, as previously described [31]. Briefly, 100 ng of genomic DNA was subjected to primary PCR amplification, followed by nested PCR. Amplified products were cloned into plasmid vectors, and individual inserts were sequenced to assess intra-patient sequence variation.

### 4.9. Estimation of Synonymous and Nonsynonymous Substitution Rates

To estimate CTL-mediated selection pressure, rates of synonymous (Ds) and nonsynonymous (Dn) substitutions within the Tax 11–19–encoding region in 18 HLA-A*02-positive HAM patients were calculated. Phylogenetic trees were constructed for each patient using the maximum likelihood method, and ancestral nucleotide sequences were inferred at each node using the PAML software package (version 4.0) [48]. For each patient, the total number of synonymous (Ns) and nonsynonymous (Nn) substitutions was determined, and the average number of synonymous (Ss) and nonsynonymous (Sn) sites per codon was calculated [49]. Ds and Dn were then defined as Ds = Ns/Ss and Dn = Nn/Sn, respectively. At positions 4, 5, 6, and 8 of the Tax 11–19 epitope, patients were stratified into high or low TCR degeneracy groups based on the mean relative recognition of the corresponding APL. Dn-Ds values were subsequently calculated for each group at each respective position.

### 4.10. Detection of CTL Responses to Naturally Occurring Variant Epitopes

Five naturally occurring HTLV-1 Tax 11–19 epitope variants (L2F, G4R, V7I, V7A, and V9A; Table 1), along with the cognate Tax 11–19 peptide and an HLA-A*02-restricted influenza M1 peptide, were synthesized. Subsequently, 5 × 10^5^ PBMCs from seven HLA-A*02-positive HAM patients were co-cultured with an equal number of HLA-A*02:01-transfected Hmy2.C1R cells pulsed with each peptide. The co-culture was maintained for 6 h in the presence of brefeldin A. Following cell culture, cells were stained for surface CD8 and intracellular IFN-γ, and analyzed using flow cytometry. The relative recognition of each variant epitope was calculated using the following formula:

(% relative recognition) = (Frequency of IFN-γ + CD8+ cells with variant epitope-background)/(Frequency of IFN-γ + CD8+ cells with Tax 11–19-background) × 100

Here, background refers to the frequency of IFN-γ + CD8+ T cells in the absence of peptide stimulation.

### 4.11. Statistical Analysis

Statistical analyses were conducted using appropriate non-parametric methods. The Mann–Whitney U test, with Bonferroni correction for multiple comparisons, was employed to compare the relative recognition of APLs and naturally occurring epitope variants. Spearman’s rank correlation coefficient was used to evaluate associations between TCR degeneracy and either HTLV-1 PVL or the relative recognition of naturally occurring epitope variants, as well as between TCR structural diversity and PVL. Chi-square tests were used to compare frequencies of synonymous and nonsynonymous substitutions. A *p*-value < 0.05 was considered statistically significant.

## Figures and Tables

**Table 1:** Naturally occurring variants of the HTLV-1 Tax 11–19 epitope in 18 HLA-A*02-positive HAM patients.

Patient	Proviral Load ^a^	CTL Frequency ^b^	TCR Degeneracy ^c^	Tax 11–19 Sequence ^d^	Designated Variant Name ^e^	Frequency of Variants ^f^	Percentage of Variants
H2	1877	4.9	13.6	LLFGYPVYV		45/46	
				***R*****	G4R	1/46	2.2
H3	821	0.4	2.5	LLFGYPVYV		44/45	
				********A	V9A	1/45	2.2
H6	2230	0.9	−4.7	LLFGYPVYV		47/48	
				******I**	V7I	1/48	2.1
H7	303	1.9	48.7	LLFGYPVYV		47/47	0.0
H8	454	0.6	82.1	LLFGYPVYV		47/47	0.0
H9	458	3.4	17.7	LLFGYPVYV		38/38	0.0
H10	130	0.4	16.2	LLFGYPVYV		46/46	0.0
H11	319	3.0	18.9	LLFGYPVYV		41/41	0.0
H12	646	19.5	38.0	LLFGYPVYV		46/48	
				***R*****	G4R	2/48	4.2
H13	1876	2.3	0.4	LLFGYPVYV		48/48	0.0
H14	1595	3.3	8.9	LLFGYPVYV		48/48	0.0
H15	394	0.5	16.0	LLFGYPVYV		43/48	
				***R*****	G4R	4/48	8.3
				******A**	V7A	1/48	2.1
H16	149	4.5	21.1	LLFGYPVYV		45/46	
				***R*****	G4R	1/46	2.2
H19	951	0.43	−14.0	LLFGYPVYV		44/45	
				*F*******	L2F	1/45	2.2
H20	224	0.5	94.0	LLFGYPVYV		45/45	0.0
H22	159	1.4	15.3	LLFGYPVYV		35/39	
				***R*****	G4R	4/39	10.3
H23	606	5.5	73.7	LLFGYPVYV		44/45	
				******I**	V7I	1/45	2.2
H24	1207	0.7	30.1	LLFGYPVYV		48/48	0.0

Abbreviations: CTL, cytotoxic T lymphocyte; HAM, HTLV-1-associated myelopathy; HTLV-1, human T-cell leukemia virus type 1; PBMC, peripheral blood mononuclear cell; TCR, T-cell receptor. ^a^: HTLV-1 proviral load, expressed as copies per 10^4^ PBMCs. ^b^: Frequency of HTLV-1 Tax 11–19-specific CTLs within the CD8+ cell population, expressed as a percentage. ^c^: TCR degeneracy was quantified as the percentage of the frequency of Y5A-specific CTLs relative to that of Tax 11–19-specific CTLs. ^d^: Amino acid sequences corresponding to the Tax 11–19 epitope based on patient-derived viral gene sequences. Residues identical to the prototype sequence are indicated by asterisks. ^e^: Naturally occurring epitope variants are designated according to amino acid substitutions; for example, G4R indicates substitution of glycine with arginine at position 4 of the Tax 11–19 epitope. ^f^: Variant frequency, defined as the proportion of epitope variants among total sequenced clones from each patient.

**Table 2:** Total number of nonsynonymous (Nn) and synonymous (Ns) substitutions in the HTLV-1 Tax 11–19 epitope region in patient groups stratified by TCR degeneracy.

	Nn	Ns
Substitutions in high TCR degeneracy	27	6
Substitutions in low TCR degeneracy	17	14

## Data Availability

The data presented in this study are available from the corresponding author upon reasonable request.

## Supplementary Materials

The following supporting information can be downloaded at: https://www.mdpi.com/article/10.3390/ijms26146602/s1.
