# Supplementary material for: High TCR Degeneracy Enhances Antiviral Efficacy of HTLV-1-Specific CTLs by Targeting Variant Viruses in HAM Patients
Source: Int J Mol Sci. 2025 Jul 10;26(14):6602. doi: 10.3390/ijms26146602 (PMC12295267; doi:10.3390/ijms26146602)
Supplement: Supplementary file 1 [file ijms-26-06602-s001.zip › ijms-3715047-supplementary.pdf]

Supplementary Table S1. Clinical characteristics of 24 HLA-A*02-positive HAM patients

Patient	Age	Sex*	PVL**	CTL%***
H1	58	M	504	9.9
H2	50	F	1877	4.9
H3	29	M	821	0.4
H4	60	F	422	0.5
H5	56	F	533	4.2
H6	55	M	2230	0.9
H7	45	F	303	1.9
H8	75	F	454	0.6
H9	75	F	458	3.4
H10	48	M	130	0.4
H11	62	F	319	3.0
H12	61	M	646	19.5
H13	52	F	1876	2.3
H14	63	F	1595	3.3
H15	65	F	394	0.5
H16	70	F	149	4.5
H17	57	F	182	2.94
H18	38	F	2972	1.36
H19	69	F	951	0.43
H20	53	F	224	0.5
H21	71	F	39	0.53
H22	60	M	159	1.4
H23	44	F	606	5.5
H24	58	M	1207	0.7
Mean ± SD	57.3 ± 11.3		793.8 ± 772.3	3.1 ± 4.2

*Seventeen females and seven males, **PVL: HTLV-1 proviral load (copy/10^4 PBMCs), ***CTL%: Frequency of HTLV-1 Tax 11-19-specific CD8+ T cells within the total CD8+ T cell population in PBMCs, measured by intracellular IFN-γ detection.
